# China’s top 10 breakthroughs in science and technology in 2025

**DOI:** 10.1093/nsr/nwag088

**Published:** 2026-02-09

**Authors:** Weijie Zhao, Xiaoling Yu

**Figure:**
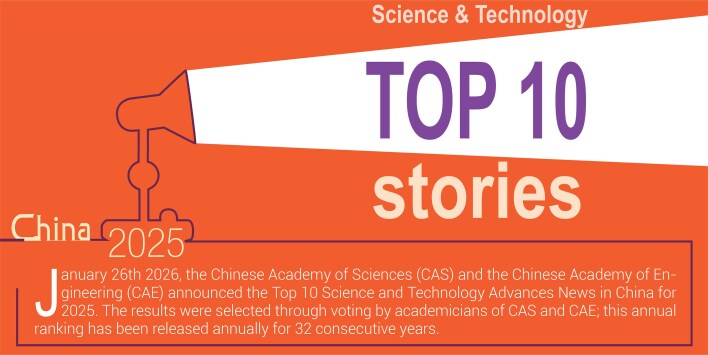


## 1 CHINESE ‘ARTIFICIAL SUN’ EAST SETS WORLD RECORD

On January 20th 2025, China’s Experimental Advanced Superconducting Tokamak (EAST), known as the Chinese ‘artificial sun,’ set a new world record in Hefei, Anhui Province. For the first time, it achieved high-quality burning at 100 million degrees Celsius for 1066 seconds. This milestone marks a significant leap of China’s fusion energy endeavor from basic research to engineering practice, and is of great importance for accelerating mankind’s realization of fusion power generation.

Shaped like a giant vessel, EAST integrates cutting-edge technologies of ultra-high temperature, ultra-low temperature, ultra-high vacuum, ultra-strong magnetic field, and ultra-high current. Its operation requires nearly 1 million components working in synergy. The EAST now holds nearly 2000 patents.

**Figure:**
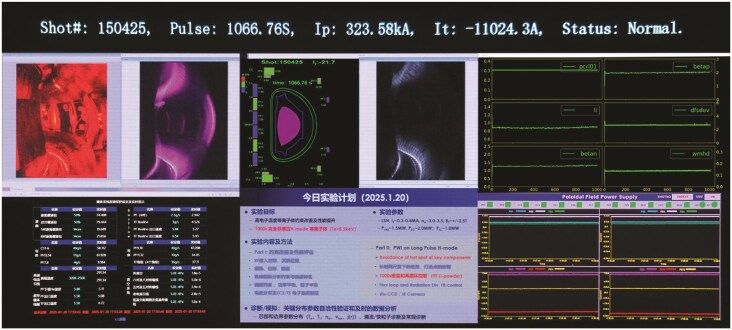
Screenshot of EAST’s world record. Image credit: Hefei Institutes of Physical Science, CAS.

Construction of the EAST project started in the early 2000s. It had undergone > 150 000 experiments before it ultimately achieved the long-pulse, high-confinement mode plasma operation at ‘100 million degrees Celsius for 1000 seconds,’ scaling new heights in fusion science.

## 2 DeepSeek: THE CHINESE AI MODEL

On January 20th 2025, DeepSeek, a company that had been founded for just over 1 year, launched its new-generation large language model, DeepSeek-R1. It achieves performance comparable to OpenAI o1 at an ultra-low training cost and is fully open source, sending shockwaves through the global artificial intelligence (AI) community. Silicon Valley’s top investor Marc Andreessen exclaimed the release of DeepSeek-R1 to be the ‘Sputnik Moment’ for AI.

**Figure:**
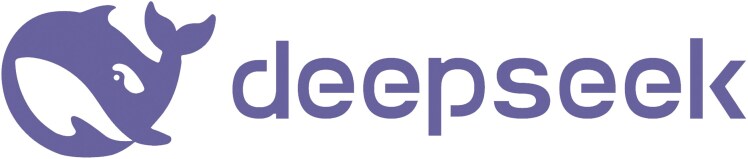
Logo of DeepSeek. Image credit: DeepSeek.

Industry insiders noted that, in a sense, the emergence of DeepSeek-R1 signals that China has evolved from following OpenAI’s lead to setting new benchmarks in AI model development.

Yongdong Zhang, Chief Scientist of the State Key Laboratory of Communication Content Cognition, stated: ‘DeepSeek-R1 represents a major breakthrough in the field of AI large models. It not only challenges OpenAI’s leading position but also injects new vitality into AI technology.’

## 3 THORIUM-BASED MOLTEN SALT REACTOR CONSTRUCTED

On November 1st 2025, CAS announced that the 2-MWt liquid-fueled thorium-based molten salt experimental reactor, which was constructed by a group led by the CAS Shanghai Institute of Applied Physics, successfully achieved thorium–uranium nuclear fuel conversion for the first time. This success makes the reactor the only currently operational molten salt reactor in the world to have implemented thorium fuel loading, and demonstrates the technical feasibility of thorium-based molten salt reactors.

Molten salt reactors are fourth-generation nuclear energy systems that use high-temperature molten salt as a coolant and are internationally recognized as the reactor type best suited for utilizing thorium resources.

**Figure:**
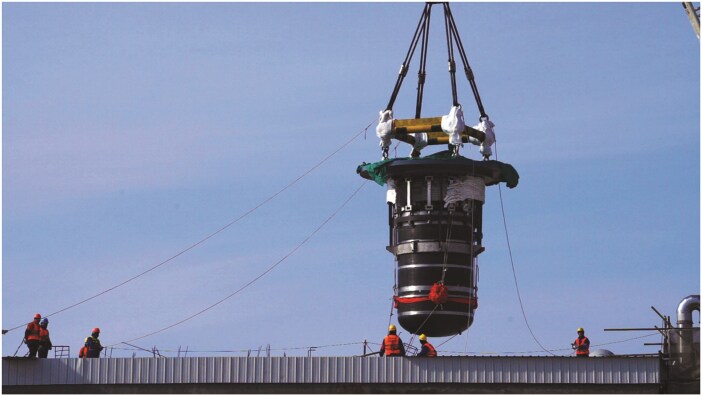
The main body of the thorium-based molten salt reactor. Image credit: the Shanghai Institute of Applied Physics, CAS.

The construction of the experimental reactor and its first successful thorium–uranium conversion lays a solid foundation for the ‘three-step’ plan: the experimental reactor, the research reactor, and the demonstration reactor. It provides strong support for China to take the lead in this field.

## 4 TIMES: THE HEPATOCELLULAR CARCINOMA PREDICTION SYSTEM

A research team led by Prof. Cheng Sun from the University of Science and Technology of China and collaborators developed a high-precision AI diagnostic tool that predicts the risk of hepatocellular carcinoma (HCC) recurrence with an accuracy rate of 82.2%. This work signifies the birth of a new research direction—computational tumor immunology, and was published in *Nature* as a cover article.

The research team discovered that the key factor determining HCC recurrence is not the number of immune cells but their location within the tumor. The density of CD57 + NK cells located at the invasive front of the tumor proved to be the strongest prognostic indicator, with a predictive accuracy far exceeding traditional methods.

Based on this discovery, the team developed the world’s first spatial immune scoring system, TIMES. This system requires only five biomarkers and conventional pathological slides to complete assessment in 12 minutes. Its cost is 90% lower than traditional sequencing methods, yet its accuracy reached 82.2%, 37% higher than the traditional TNM system. The TIMES system is now freely available for doctors and patients worldwide.

**Figure:**
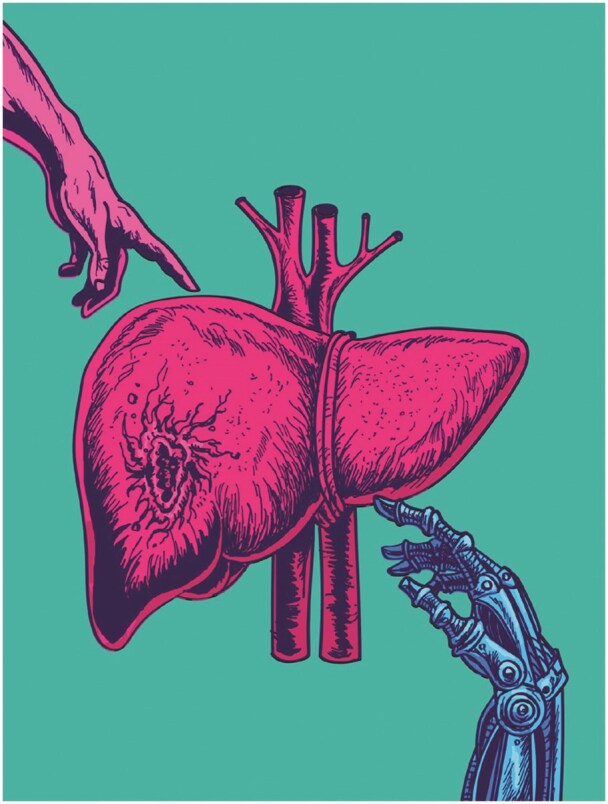
Art illustration of the HCC recurrence prediction system. Image credit: University of Science and Technology of China.

## 5 BCI SYSTEM BEINAO-1 TESTED IN PATIENTS

The semi-invasive ‘Beinao-1’ and invasive ‘Beinao-2’ intelligent brain-computer interface (BCI) systems, jointly developed by the Chinese Institute for Brain Research, Beijing and its affiliated company Beijing Xinzhida Neural Technology, both achieved world-leading standards.

On 20 March 2025, it was announced that the wireless Beinao-1 system has been successfully implanted in its first batch of patients. The patients recovered well post-surgery, with the device’s effective channel count exceeding 98%.

With the system, paralyzed patients can remotely control computers, robotic arms, and even drive muscle stimulation devices to promote the recovery of their own limb motor functions. In another case, as the world’s first wireless fully-implantable BCI system with Chinese language decoding capability, Beinao-1 successfully helped a patient who lost speech due to amyotrophic lateral sclerosis (ALS) to restore the ability to communicate.

**Figure:**
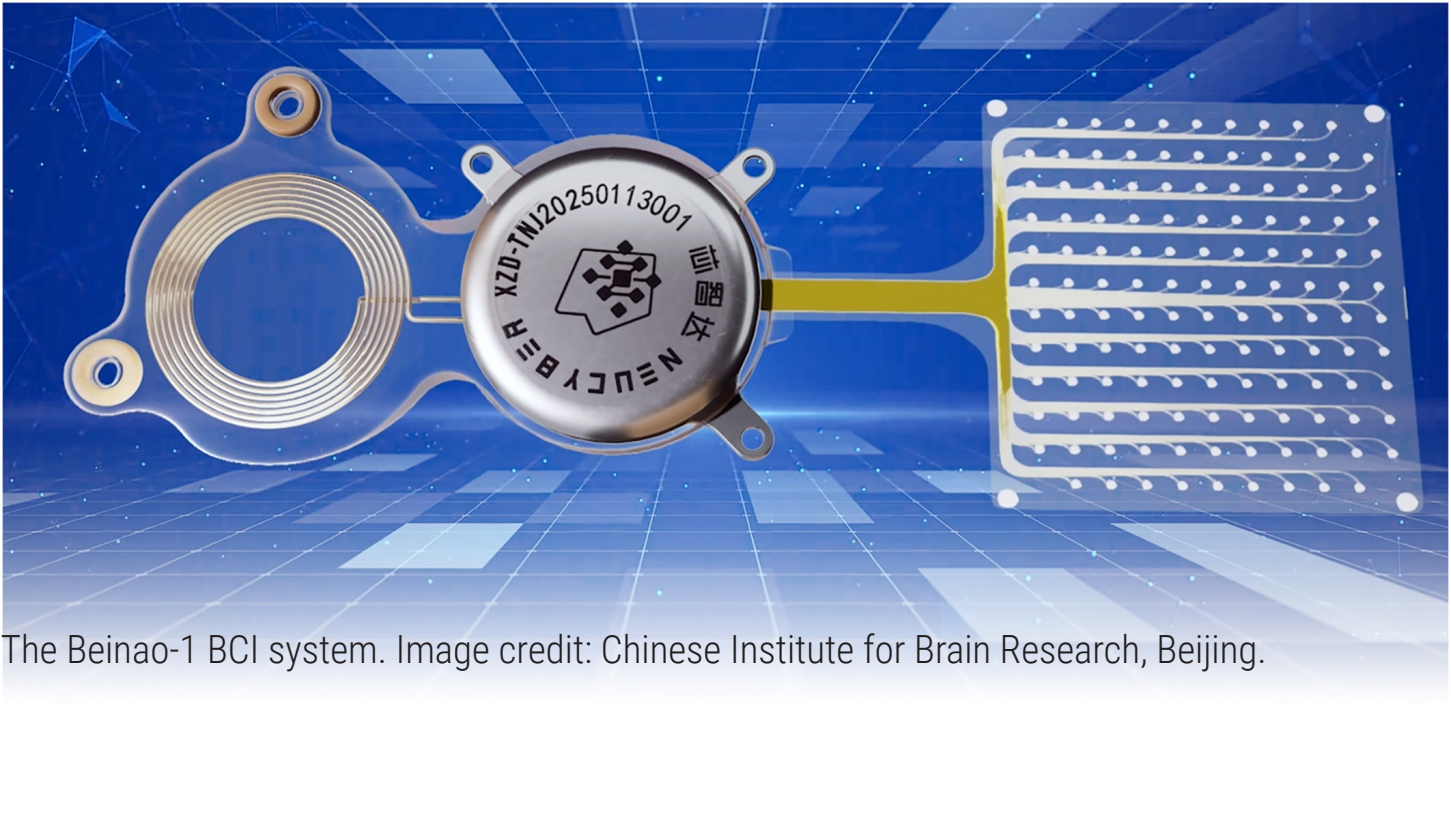
The Beinao-1 BCI system. Image credit: Chinese Institute for Brain Research, Beijing.

## 6 NOVEL TARGET AND CANDIDATE DRUG FOR PARKINSON’S DISEASE

A group led by Profs. Jintai Yu and Peng Yuan from Fudan University, in collaboration with Prof. Cong Liu’s team from the CAS Interdisciplinary Research Center on Biology and Chemistry, revealed that the previously functionally unknown gene *FAM171A2* is a key molecule promoting the occurrence and progression of Parkinson’s disease (PD). They also screened out small-molecule compounds with potential therapeutic value, bringing new hope for slowing PD progression. This research was published in *Science* on 21 February 2025.

This work not only opens new directions for PD drug development but also brings new hope to millions of patients worldwide. With further in-depth research and clinical endeavors, *FAM171A2* is expected to become a key target in combating PD.

**Figure:**
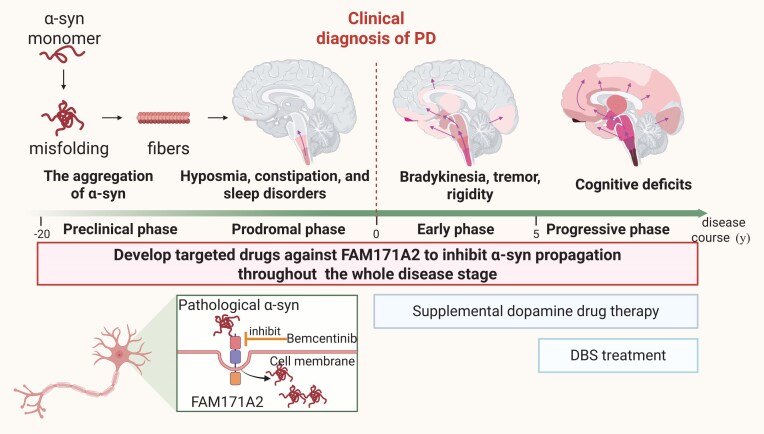
New drugs targeting *FAM171A2* bring new hope to PD patients. Image credit: Fudan University.

## 7 SUPERCONDUCTING QUANTUM COMPUTING PROTOTYPE: ZUCHONGZHI-3

Jian-Wei Pan, Xiaobo Zhu, Cheng-Zhi Peng and coworkers from the University of Science and Technology of China successfully constructed the superconducting quantum computing prototype Zuchongzhi-3, establishing a new benchmark in quantum computational advantage. The results were published in *Physical Review Letters* on 3 March 2025. A reviewer described it as ‘benchmarking a new superconducting quantum computer, which shows state-of-the-art performance.’

Zuchongzhi-3 contains 105 qubits and 182 couplers, with multiple key performance indicators significantly improved. To evaluate its capabilities, the team conducted an 83-qubit, 32-layer random circuit sampling task on the system. Compared to the current optimal classical algorithm, the computational speed surpasses that of the world’s most powerful supercomputer by 15 orders of magnitude. It also outperforms the latest results published by Google in October 2024 by 6 orders of magnitude, establishing the strongest quantum computational advantage in a superconducting system to date.

The research team is now working towards areas such as quantum error correction, quantum entanglement, quantum simulation, and quantum chemistry.

**Figure:**
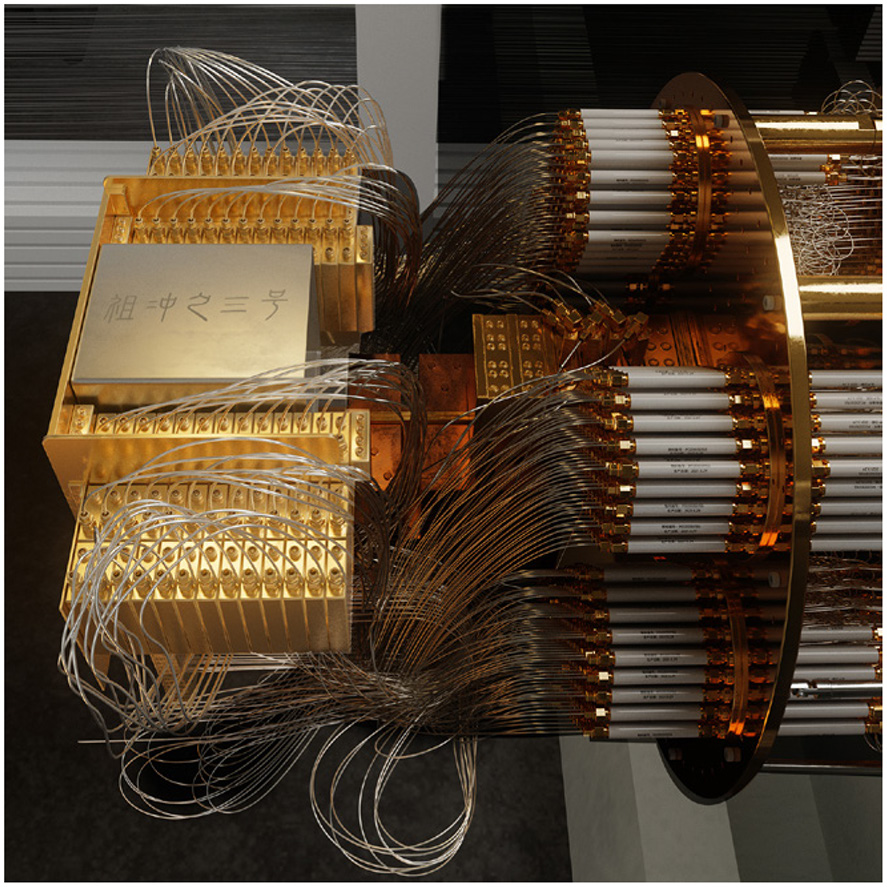
The cryogenic testing system for Zuchongzhi-3. Image credit: University of Science and Technology of China.

## 8 NEW HYDROGEN PRODUCTION PATHWAY

A team led by Prof. Ding Ma from Peking University and collaborators achieved major breakthroughs in hydrogen production, with the results published in *Nature* on 13 February 2025, and in *Science* on 14 February 2025.

Both studies aimed to optimize hydrogen production reactions but differed significantly in research focus and reaction pathways. The work published in *Nature* broke through the stability bottleneck. It innovatively introduced rare earth elements to develop a universally applicable strategy for stabilizing highly active hydrogen production catalysts. The work published in *Science* created a zero-carbon emission hydrogen production pathway via ethanol and water molecule reforming, laying a solid scientific foundation for industrial zero-carbon hydrogen production.

**Figure:**
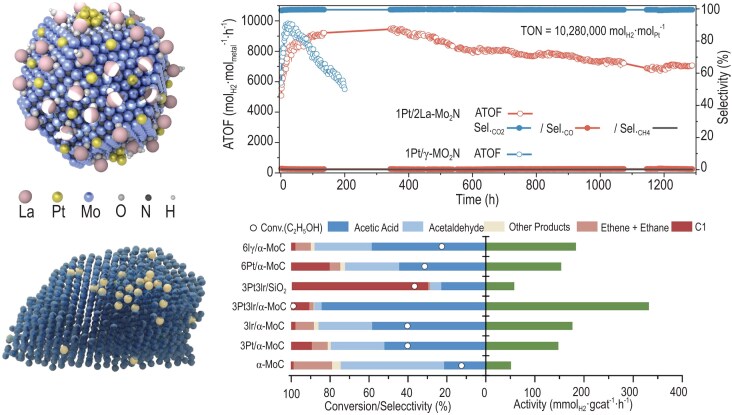
The new hydrogen production pathway. Image credit: Peking University.

Through novel catalysts, the newly developed high-yield hydrogen production pathway successfully eliminates carbon dioxide emissions at the source.

## 9 SAFEGUARG THE 'BLACK SOIL GRANARY'

**Figure:**
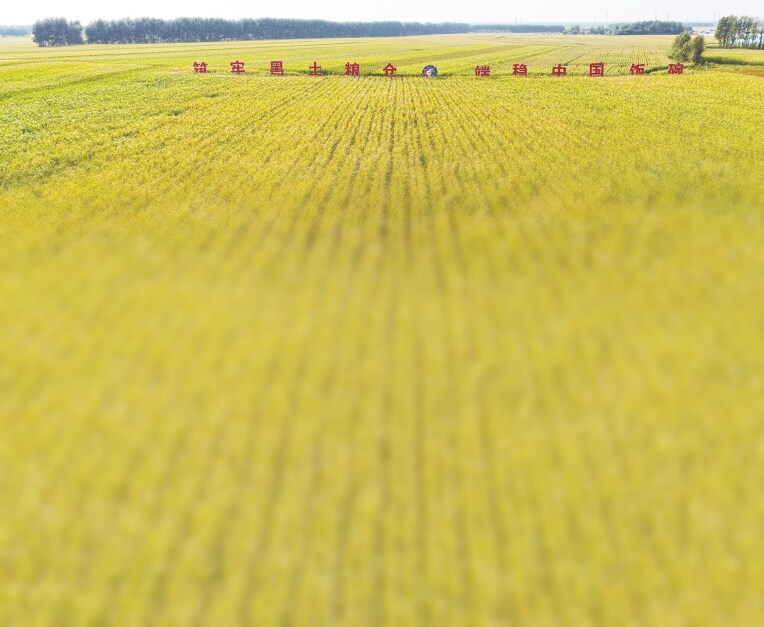
The ‘Black Soil Granary’ Scientific and Technological Initiative achieved major breakthroughs. Image credit: Northeast Institute of Geography and Agroecology, CAS.

The fertile black soil plains in northeastern China make it one of the country’s vital bases for agricultural production. Collaborating with the northeastern provinces, CAS started the ‘Black Soil Granary’ Scientific and Technological Initiative in 2021. The initiative gathered over 90 institutions to safeguard the sustainable utilization of the ‘black soil granary.’

On the annual work conference of the initiative held on 9 April 2025 in Harbin, Heilongjiang Province, major achievements of the initiative were released: an integrated sky–land monitoring technology system has been established; China’s first set of remote sensing maps for soil carbon and nitrogen in typical black soil regions at a 10-meter spatial resolution was completed; theories for black soil degradation control and healthy cultivation were proposed; a comprehensive slope-gully erosion prevention and control technology system was developed; a soybean intelligent breeding technology system was established; the Dongsheng soybean cultivar series, especially the new cultivar Dongsheng 22, has been widely promoted and grown; technologies such as the Honghu series of intelligent agricultural machinery and the Fuxi smart agricultural management system have promoted the rapid development of smart agriculture; and a series of replicable agricultural models for black soil areas have been explored and summarized.

## 10 OPTOELECTRONIC INTEGRATED SYSTEM FOR 6 G COMMUNICATION

A joint team from Peking University and the City University of Hong Kong successfully developed an ultra-broadband optoelectronic integrated system for 6 G communication. The system achieved full-frequency-band, flexibly tunable high-speed wireless communication for the first time. The results were published online in *Nature* on 27 August 2025.

The system achieves high-speed transmission at any frequency within the wireless signal range of 0.5–115 GHz. This full-frequency-band compatibility is internationally leading. The system is flexibly tunable, allowing it to switch to a secure frequency band and establish a new channel when signals are interfered with. Thus, the reliability and spectrum utilization efficiency are significantly enhanced.

In the future, the system can integrate with AI algorithms to generate wireless networks that are more flexible and intelligent. These networks will be much safer with the ability to automatically avoid interference, and will enable real-time data transmission and precise environmental sensing in various complex scenarios.

**Figure:**
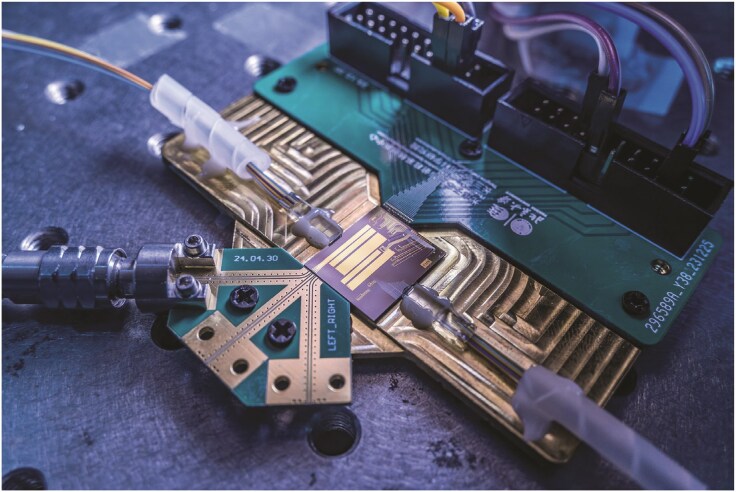
The ultra-broadband optoelectronic integrated chip. Image credit: Peking University.

